# The Role of MicroRNAs Carried by Extracellular Vesicles in Tumorigenesis Through Reprogramming the Mitochondrial Information Processing System

**DOI:** 10.3390/ijms27115112

**Published:** 2026-06-05

**Authors:** Arpita Ghosh-Mitra, Mansi Patel, Samarjit Das

**Affiliations:** 1 Rajiv Gandhi Cancer Institute and Research Centre, New Delhi 110085, India; arpitaghoshmit@gmail.com (A.G.-M.); mansipatel241002@gmail.com (M.P.); 2 Department of Anesthesiology & Critical Care Medicine, Johns Hopkins School of Medicine, Baltimore, MD 21205, USA

**Keywords:** extracellular vesicles, EV, exosomes, microRNA, miRNA, mitochondria, cancer

## Abstract

Mitochondrial dysfunction is not merely a byproduct of transformation but a driver of tumorigenesis, metastasis, and therapeutic resistance. Recent advancements in intercellular communication have identified Extracellular Vesicles (EVs) or exosomes as critical mediators that bridge the gap between the tumor and its microenvironment (TME). These EVs contain a complex repertoire of bioactive cargo, including proteins, lipids, and RNAs. Among the class of RNAs, small non-coding RNAs, microRNAs (miRNAs), are the most abundantly expressed bioactive compounds that are selectively packaged and delivered to recipient cells. EV-delivered miRNAs can target nuclear-encoded mitochondrial genes and have also been reported to localize to mitochondria (mitomiRs), where they function as post-transcriptional regulators of bioenergetic and mitochondrial dynamic adaptations that support tumor progression. This review explores the “EV-miRNA-Mitochondria Axis”, delineating the molecular mechanisms by which EV-carried miRNAs reprogram the “Mitochondrial Information Processing System” (MIPS) - a signaling network where mitochondria integrate metabolic cues (e.g., ROS, calcium flux) to dictate critical biological outcomes, such as immune regulation and cell survival. We summarized specific sorting machineries (e.g., hnRNPA2B1, Lupus La) that package oncogenic miRNAs into EVs and how these cargoes hijack mitochondrial function upon delivery. Specifically, we discussed how EV-miRNAs induce metabolic shifts, manipulate mitochondrial dynamics (fission/fusion), and inhibit the intrinsic apoptosis to drive cancer progression. Finally, we highlighted the dual utility of these EV-miRNAs as drivers of pathogenesis and promising non-invasive biomarkers for early diagnosis, prognostic and therapeutic monitoring.

## 1. Background

### 1.1. The Extracellular Vesicle Landscape and Cargo Diversity

Tumorigenesis is a systemic process that relies heavily on the crosstalk among cancer cells and various stromal cells in the tumor microenvironment (TME) [1]. Extracellular Vesicles (EVs) play an important role in this cell-to-cell communication. EVs are heterogeneous, lipid bilayer-enclosed structures released by almost every cell type, including tumor cells, mesenchymal stem cells, and immune cells, to maintain their regular cellular homeostasis. They are broadly categorized based on their biogenesis into small EVs (sEVs or exosomes, 30–150 nm), which originate from the endosomal system, and larger vesicles, often classified as medium/large EVs (>200 nm), which include microvesicles shed from the plasma membrane and apoptotic bodies released by dying cells [2]. While microvesicles typically range from 100 nm to 1 µm, apoptotic bodies represent the largest subclass, often exceeding 1 µm in diameter [3,4].

Contrary to early views of EVs as mere cellular waste disposal units, they are now recognized as well-regulated “signalosomes” capable of transferring a diverse array of bioactive cargo. This cargo includes proteins (e.g., enzymes, signaling receptors, and cytoskeletal components), lipids (e.g., ceramides and sphingomyelin), and nucleic acids (mRNA and non-coding RNAs) [3]. While the presence of genomic DNA in small EVs remains a subject of debate, tumor-derived EVs and larger vesicle subtypes have been shown to transfer significant amounts of mitochondrial DNA [4,5]. Recent evidence even suggests that EVs can transport whole functional mitochondria or mitochondrial DNA (mtDNA) to rescue bioenergetics in recipient cells [6,7]. However, among these transferred molecules, microRNAs (miRNAs) have emerged as particularly potent regulators of recipient cell function [8].

### 1.2. Mechanisms of Selective miRNA Sorting

MicroRNAs are short (19–24 nucleotides) non-coding RNAs that regulate gene expression post-transcriptionally. When packaged into EVs, miRNAs are shielded from extracellular RNase degradation, allowing them to translocate stably through the circulation and exert effects at distant sites [8]. Importantly, the loading of miRNAs into EVs is not a random process but a highly selective one regulated by specific cellular machineries. RNA-binding proteins (RBPs) such as hnRNPA2B1 and SYNCRIP recognize specific motifs (EXOmotifs) on miRNAs to guide their sorting into EVs [3]. Additionally, the RNA-binding protein Lupus La has been identified as a key mediator in the selective packaging of specific miRNAs, such as miR-122, in breast cancer cells [3,9]. This selective packaging ensures that specific oncogenic or tumor-suppressive signals are transmitted to target cells, allowing the donor tumor cell to manipulate its environment remotely.

### 1.3. Alterations of Mitochondrial Function During Tumorigenesis

Once internalized by recipient cells, EV-miRNAs function as critical effectors of the “Mitochondrial Information Processing System” (MIPS) [1]. Mitochondria are no longer viewed solely as powerhouses but as important signaling hubs that transduce biological inputs into outputs determining cellular function, metabolic state, and survival [8]. In neoplasia, mitochondrial dysfunction is a hallmark of tumor progression. EV-miRNAs hijack mitochondrial biology through three primary mechanisms.

#### 1.3.1. Metabolic Reprogramming

Cancer cells utilize EV-miRNAs to enforce metabolic plasticity. For instance, tumor cell-derived EVs carrying miR-210 target iron-sulfur cluster assembly proteins (ISCU), disrupting the electron transport chain (ETC) and forcing recipient cells into aerobic glycolysis (the Warburg effect) [3]. Conversely, cancer cells can reprogram stromal fibroblast-derived EVs carrying specific miRNAs, such as miR-155 and miR-210, thereby inducing the “Reverse Warburg Effect,” whereby stromal glycolysis supplies energy-rich metabolites that support tumor mitochondrial oxidative phosphorylation (OXPHOS) [9,10,11].

#### 1.3.2. Regulation of Mitochondrial Dynamics

The balance between mitochondrial fission and fusion is vital for tumor metastasis. EV-miRNAs can target key dynamic regulators to manipulate this balance; for instance, cancer-associated fibroblast (CAF)-derived EVs transfer miR-106b to target Mitofusin-2 (Mfn2). The resulting loss of Mfn2 inhibits mitochondrial fusion, driving a fragmented mitochondrial network that enhances tumor invasiveness and chemoresistance [9].

#### 1.3.3. Evasion of Apoptosis

The intrinsic apoptotic pathway is governed by mitochondrial outer membrane permeabilization (MOMP), regulated by the Bcl-2 family protein. EV-miRNAs confer chemoresistance by disrupting this balance. These apoptosis-modulating EVs typically originate either from drug-resistant tumor cells which transfer their survival phenotype to neighboring, drug-sensitive tumor cells or from stromal cells within the tumor microenvironment. For example, cancer-associated fibroblast (CAF)-derived EV-miR-21 to neighboring cancer cells suppresses APAF1, inhibiting apoptosis, and inducing paclitaxel resistance [12]. EVs secreted from breast cancer stem cells (CDCs) and chemoresistant cells express a high level of miR-155. miR-155 upregulation in chemoresistant cells associated with epithelial-to-mesenchymal transition indicates transfer of EV-miR-155 from CDCs and chemoresistant cells [13]. Another example of stromal cells transferring miRNA to liver cancer cells is when Zhang et al. [14] revealed a significant downregulation of miR-320a level in CAF-derived EVs that affect cancer cell proliferation, migration and metastasis. The authors have also validated that miR-320a in liver cancer cells directly targets the 3′-UTR of PBX3 mRNA, which in turn inhibits the activation of the MAPK pathway. It has already been demonstrated that intercellular crosstalk between tumor cells and fibroblasts is mediated by tumor-derived EVs. High-metastatic liver cancer cells secrete EV-encapsulated miR-1247-3p that directly targets B4GALT3, leading to activation of β1-integrin-NF-κB signaling in fibroblasts. Activated CAFs further promote lung metastasis of liver cancer [15].

## 2. Mechanisms of EV Biogenesis, Cargo Sorting, and Functional Delivery

To understand how cancer cells utilize EV biogenesis to manipulate mitochondrial function remotely, it is necessary to deconstruct the molecular machinery responsible for processing these vesicles and, more importantly, the specific “codes” and RBPs that dictate which miRNAs are exported. The composition of EVs is not a passive reflection of the donor cell’s cytoplasmic bioactive compounds; rather, it is the result of a highly regulated intracellular sorting system that creates a distinct “molecular fingerprint” designed to alter the phenotype of the recipient cell [16,17].

### 2.1. The Heterogeneity of EV Biogenesis

EVs are a heterogeneous population of particles, and their biogenesis dictates their protein and lipid composition, which in turn influences their ability to target mitochondrial function. Broadly, these EVs are generated through three distinct pathways: the endosomal pathway (generating small EVs or exosomes), outward budding of the plasma membrane (generating microvesicles/ectosomes), and cell fragmentation during programmed cell death (generating apoptotic bodies) [4,18]. However, in the context of active intercellular communication within the tumor microenvironment, research primarily focuses on the exosomal and plasma membrane budding pathways, as these are utilized by living cells to selectively package and deliver functional cargo.

#### 2.1.1. The Endosomal Pathway (Small EVs or Exosomes)

Small EVs (30–150 nm) originate within the endosomal system. The biogenesis process begins with the invagination of the limiting membrane of late endosomes, forming intraluminal vesicles (ILVs) within multivesicular bodies (MVBs). This inward budding is a critical checkpoint for cargo sorting. It is driven primarily by the Endosomal Sorting Complex Required for Transport (ESCRT) machinery, comprising four distinct complexes (ESCRT-0, -I, -II, and -III) and associated proteins like ALIX and TSG101, which recognize ubiquitinated proteins and cluster them into the forming intercellular vesicles [19]. However, tumor cells frequently utilize ESCRT-independent pathways to bolster EV secretion under stress. One such mechanism involves ceramides. The enzyme neutral sphingomyelinase 2 (nSMase2) hydrolyzes sphingomyelin into ceramide, which spontaneously creates lipid raft-enriched microdomains that induce membrane curvature and ILV formation. This lipid-based pathway is particularly relevant to mitochondrial regulation because nSMase2 activity has been directly linked to the secretion of miRNAs such as miR-210 [19,20]. Upon transfer, EV-miR-210 promotes angiogenesis in recipient endothelial cells by targeting Ephrin-A3, and it simultaneously drives metabolic reprogramming in stromal cells by targeting specific mitochondrial respiration enzymes, including NDUFA4 (Complex I subunit), SDHD (Complex II subunit), and ISCU (Iron-Sulfur Cluster Assembly Enzyme) [3]. While historically categorized strictly by size, recent high-resolution iodixanol density gradient fractionation has revealed that cancer cells, such as metastatic breast cancer cells (MDA-MB-231), release distinct subpopulations of small EVs that share similar sizes (~110–120 nm) but possess fundamentally different biophysical properties and cargo sorting mechanisms [21]. The subpopulations of small EVs are as follows:

- Vesicular Low Density (vLD): This subpopulation floats at a density of 1.09–1.11 g/mL. vLD EVs are enriched in CD9, a tetraspanin protein associated with the plasma membrane, but lack endosomal markers. This suggests that despite their small size, vLDs likely originate from the plasma membrane (microvesicles/ectosomes) rather than the endosomal system [11,19,20].
- Vesicular High Density (vHD): This subpopulation floats at a density of 1.14–1.16 g/mL and is highly enriched in CD63 and endosome-associated proteins (e.g., syntenin-1), consistent with a classical exosomal origin [11,21,22].

These subpopulations exhibit distinct RNA sorting behaviors—the vLD EV population appears to package miRNAs non-selectively, meaning the miRNA abundance in these EVs essentially mirrors the cytosolic concentration of the donor cell. In contrast, the vHD EV population displays selective active sorting, enriching specific miRNAs (such as miR-122) far beyond their cellular stoichiometric ratios. This distinction is vital for understanding tumorigenesis, as it implies that specific “high-occupancy” EVs (vHD) are engineered by the tumor cell to deliver potent signaling payloads, while others (vLD) may serve different communicative or waste-disposal functions [11].

#### 2.1.2. The Plasma Membrane Pathway (Microparticles or Microvesicles)

In contrast to small EVs, microvesicles or microparticles (100–1000 nm) are generated by the direct outward budding and fission of the plasma membrane [23]. This process is regulated by cytoskeletal remodeling proteins, such as ARF6 and RhoA, and changes in lipid asymmetry, specifically the exposure of phosphatidylserine (PS) on the outer leaflet. The formation of microvesicles is triggered by the rearrangement of the actin cytoskeleton, regulated primarily by the small GTPases RhoA and ARF6. For instance, the activation of RhoA triggers a specific signaling pathway involving Rho-associated kinase (ROCK) and LIM kinase, which is essential for the generation of transforming microvesicles in cancer cells [24]. Simultaneously, ARF6 initiates a signaling cascade that recruits Exportin-5 to the plasma membrane, linking cytoskeletal remodeling with the targeted export of specific RNA cargoes [25]. Microvesicle shedding is heavily influenced by intracellular calcium (Ca2+) levels. Elevated Ca2+ activates lipid scramblases and inhibits flippases, leading to the loss of membrane asymmetry and the exposure of PS on the outer leaflet [23]. This lipid redistribution physically aids in generating the membrane curvature necessary for outward budding.

Unlike the endosomal sorting of small EVs, microvesicle cargo is sampled directly from the cytoplasm during budding; however, this process is not entirely passive. Specific post-translational modifications can direct RNA-binding proteins to the plasma membrane. For example, under oxidative stress, the phosphorylation of Caveolin-1 (Cav-1) leads to its interaction with the RNA-binding protein hnRNPA2B1. This complex selectively packages specific miRNAs, such as miR-17 or miR-93, into shedding microvesicles, a mechanism distinct from exosomal sorting [26]. Additionally, because they encompass cytoplasmic constituents, microvesicles have been identified as key transporters of mitochondrial proteins and even whole mitochondrial fragments, which is particularly relevant for the transfer of metabolic traits and inflammation in the nervous system and cancer [27].

### 2.2. The Molecular Codes for Selective miRNA Sorting

The most contentious and critical aspect of EV biology is determining how specific miRNAs are selected for export into the EVs while others are retained. This selectivity indicates the existence of cellular machinery dedicated to packaging specific “messages” into the EVs. Current research highlights four primary mechanisms governing this sorting process: RNA-binding proteins (RBPs), specific sequence motifs, post-transcriptional modifications, and lipid interactions [21].

#### 2.2.1. RNA-Binding Proteins (RBPs) and EXOmotifs

Specific RBPs act as chaperones, recognizing short nucleotide sequences termed EXOmotifs (which frequently feature a high G and C content) within miRNAs and shuttling them into forming EVs [28].

The heterogeneous nuclear ribonucleoprotein A2B1 (hnRNPA2B1): hnRNPA2B1 is a key player in selectively inserting miRNAs into the EVs. It binds miRNAs containing the GGAG motif, such as miR-198 and miR-601, as well as those with AGG/UAG motifs, such as miR-93 and miR-17 [29]. The sorting function of hnRNPA2B1 is regulated by sumoylation. It has been shown that only sumoylated hnRNPA2B1 encapsulates miRNAs into EVs [17].

Synaptotagmin-binding, cytoplasmic RNA-interacting protein (SYNCRIP): SYNCRIP is also known as hnRNPQ. It utilizes a specific NURR (N-terminal unit for RNA recognition) domain to recognize a G-rich motif (GGCU) at the 3′ end of miRNAs, such as miR-3470a and miR-194-2-3p, facilitating their loading into hepatocyte-derived EVs [15,29].

Lupus La Protein: Even though several studies have claimed miR-122 is a hepatocyte-enriched miRNA [30], it has been demonstrated that the selective sorting using Lupus La protein for the packaging of miR-122 in breast cancer cells. miR-122 contains two specific motifs, the UUU sequence at the 3′ end and the 5′ UGGA motif, that allow high-affinity binding to Lupus La. This interaction creates a sorting complex that is specifically directed into the vHD subpopulation of EVs, which are known to promote metastasis by reprogramming glucose metabolism in niche cells [21].

Y-Box Protein 1 (YBX1): This RBP interacts with several types of ncRNAs, including tRNAs and Y-RNAs, and is essential for the packaging of miR-223 into EVs produced by HEK293T cells. It has been proposed that YBX1 is often secreted in EVs in a non-phosphorylated state, suggesting that YBX1dephosphorylation sorts miR-217 into the EVs [21,31].

Fragile X mental retardation 1 (FMR1): This RBP controls the selective loading of specific miRNAs, such as miR-155, by recognizing the AAUGC sequence motif during inflammatory responses [29].

FUS and Alyref: These two RBPs recognize the highly enriched EXOmotif CGGGAG, acting synergistically to export their bound miRNAs into the extracellular space [29].

There is currently a lack of comprehensive, quantitative head-to-head studies establishing whether any single cargo sorting pathway predominates under specific conditions such as hypoxia or therapeutic stress. Instead, the available evidence supports a highly dynamic and context-dependent process in which distinct stressors engage multiple, partially overlapping sorting mechanisms. For example, while classical RNA-binding proteins like hnRNPA2B1 and SYNCRIP are well documented, cells exposed to noxious stimuli can alternatively utilize Caveolin-1 (CAV1) to selectively sort miRNAs into vesicles [8]. Similarly, cellular stress can trigger reversible binding between the RNA-binding protein HuR and specific miRNAs (such as miR-122), actively augmenting their extracellular export to manage the stress response. Other alternative mechanisms, such as ceramide-dependent secretion and Ago2-associated loading, also reflect the complex regulation of EV cargo under varying microenvironmental pressures [8]. Ultimately, EV-miRNA sorting under stress does not appear to rely on one universal pathway, but rather on a flexible, stimulus-specific network of sorting machineries.

#### 2.2.2. Post-Transcriptional Modifications (3′-End Remodeling)

Post-transcriptional modifications at the 3′-end of the miRNAs can facilitate the miRNA to enter the multivesicular body, determining whether the miRNA is secreted from the cells or retained in the cells [2]. Several studies have indicated that 3′-end uridylation functions as a specific sorting signal that directs miRNAs into the EVs, as evidenced by the significant enrichment of uridylated isoforms in B cell-derived and urinary EVs compared to their donor cells [11,29]. This modification is catalyzed by specific enzymes, including Terminal Uridylyl Transferase 1 (TUT1) and Zinc finger CCHC domain-containing protein 6 (ZCCHC6), which append non-templated uracil residues to the miRNA 3′-end to facilitate its export [32]. Conversely, 3′-end adenylation generally acts as a signal for cellular retention, with adenylated miRNA isoforms being predominantly sequestered within the cellular cytoplasm rather than secreted [11]. These findings suggest that 3′-end remodeling acts as a molecular “zip code,” potentially altering the miRNA’s structural or hydrophobic properties to guide its interaction with the sorting machinery during intraluminal vesicle formation [11].

#### 2.2.3. Lipid Interactions: The Ceramide and Lipid Raft Pathway

Lipids are not merely structural components of the vesicle membrane; they actively participate in EV cargo selection and EV formation [33]. Several pathways have been identified where lipid interaction plays a significant role in EV biogenesis [34].

The Neutral sphingomyelinase 2 (nSMase2)-Ceramide Pathway: nSMase2 converts sphingomyelin into ceramide. Ceramide spontaneously self-associates to form lipid raft-enriched microdomains that induce the membrane curvature required for vesicle budding from the cell membrane. This pathway is critical for the secretion of specific miRNAs, such as miR-210, which regulates angiogenesis and the hypoxic response [29,35]. Furthermore, inhibition of nSMase2 using GW4869 reduces the export of some specific miRNAs from the cells [3].

Lipid Raft Affinity: It has been identified that certain RNA transcripts, including miRNAs, possess high affinity to bind lipid rafts (cholesterol-rich microdomains) within the multivesicular body membrane [36]. Specifically, recent bioinformatics analyses have identified distinct ‘raft RNA motifs’ in EV-miRNAs that facilitate their direct interaction with lipid rafts, thereby mediating their loading into EVs independently of the ESCRT protein machinery [36].

Caveolin-1 Regulation: It has been proposed that Caveolin-1 (CAV1), a major component of lipid rafts (caveolae), regulates miRNA sorting triggered by oxidative stress [26]. Additionally, phosphorylated CAV1 promotes the O-GlcNAcylation of hnRNPA2B1, which in turn increases the protein’s affinity for specific miRNAs, such as miR-17 and miR-93, and encapsulates them into the multivesicular body [3,29].

### 2.3. Mechanisms of EV Cellular Uptake and Cargo Delivery

Once released into the extracellular space, a vast majority of EVs are rapidly cleared from circulation and excreted through biofluids such as urine [37]. However, for those that act as intercellular messengers, they must successfully dock and transfer their cargo to recipient cells to exert functional changes in the tumor microenvironment [35,38]. The uptake of EV-cargo is not a passive event but involves specific interactions between surface ligands on the EV (e.g., tetraspanins CD9, CD63, integrins) and receptors on the plasma membrane of the target cell [39,40,41]. Several mechanisms of the EV-cargo internalization have been identified, which are described as follows:

Endocytosis: This is the most common molecular pathway, involving clathrin-dependent, caveolin-dependent, or lipid raft-mediated uptake of EV cargo into the recipient cells [19]. This process is highly cell-type dependent and utilizes distinct pathways, including clathrin-mediated endocytosis, caveolin-dependent uptake, and lipid raft-mediated internalization [41,42,43]. Additionally, larger volumes of EVs can be internalized via macropinocytosis (membrane ruffling), while specialized immune cells, such as macrophages and dendritic cells, often utilize phagocytosis to engulf vesicles [41]. The uptake process is rarely passive; it is frequently initiated by specific interactions between surface proteins, such as Heparan Sulfate Proteoglycans (HSPGs), integrins, and tetraspanins, which facilitate the initial tethering of EVs to the recipient cell surface [40,44]. Once internalized, EVs are typically directed to lysosomes for degradation, though a fraction can undergo back-fusion with the endosomal membrane to release their functional RNA and protein cargo into the cytoplasm [19].

Membrane Fusion: It has also been demonstrated that the EVs can fuse directly with the plasma membrane of the recipient cell, followed by releasing their cargo immediately into the cytosol of the recipient cells. This mechanism is favored in the acidic microenvironment, which is often found in tumor cells [19]. While generally less common than endocytosis in physiological conditions, direct membrane fusion is significantly enhanced in acidic microenvironments (low pH), a hallmark of hypoxic tumor tissues [45]. Studies have demonstrated that the low pH conditions typical of the tumor microenvironment increase the rigidity of EV lipid membranes and facilitate fusion events that are otherwise rare at neutral physiological pH [45]. This fusion process may be regulated by specific lipid compositions and membrane proteins, including SNARE complexes and Rab GTPases, which facilitate the merging of the lipid bilayers [19].

Phagocytosis: Specialized immune cells like macrophages can engulf circulating EVs via phagocytosis; cancer cells exploit this uptake mechanism to deliver immunosuppressive miRNAs, such as miR-21, into immune cells, thereby modulating the tumor microenvironment to favor tumor growth and immune evasion [46,47].

Ligand-Receptor interaction: EV internalization or surface signaling is frequently mediated by specific combinations of ligands on the EV surface and receptors on the target cell membrane. For example, specific integrin complexes on tumor-derived EVs dictate organotrophic metastasis by binding to resident receptors in specific tissues (e.g., EV-integrin binds to Kupffer cells in the liver, whereas and bind to lung resident fibroblasts) [40]. Another critical example is immune evasion mediated by the PD-L1/PD-1 axis; tumor-derived EVs expressing programmed death-ligand 1 (PD-L1) bind directly to the PD-1 receptor on the surface of cytotoxic T-cells, inhibiting T-cell activation without the EV needing to be fully internalized [38]. Additionally, cellular uptake of EVs is heavily reliant on the binding of EV surface proteins to Heparan Sulfate Proteoglycans (HSPGs) and scavenger receptors (such as SR-B1) on recipient cells, as blocking these receptors significantly reduces EV internalization [19].

#### The Stoichiometry Debate and Functional Relevance

A significant controversy exists in the field concerning the stoichiometry of miRNAs per EV. Quantitative analyses suggest that on average, there is less than one specific miRNA copy per single EV, implying that a large number of EVs would be required to induce functional change in the recipient cells [48]. However, a recent study using high-resolution iodixanol density gradient centrifugation demonstrates the existence of specific EV sub-populations, such as the vHD EV, that are highly enriched with a specific miRNA, essentially acting as “high-occupancy” delivery vectors [35]. Furthermore, the continuous secretion and accumulation of these EVs in the acidic and hypoxic TME concentrate these signals locally, allowing even low-abundance miRNAs to reach the cumulative threshold levels required to repress mitochondrial function in recipient cells [20,49]. In mammalian cells, approximately 100–1000 copies of a specific miRNA per cell are needed to measurably reduce target-mRNA levels. Assuming uptake efficiencies of 0.1–1% of EV cargo and <1 functional miRNA per EV, the number of EVs that must be internalized per cell to reach this threshold. Once internalized, EV-miRNAs are functional; translocated miRNAs can be directed to specific cellular organelles in the recipient cells [50]. For example, EV cargo internalized via endocytosis can be translocated to the endoplasmic reticulum or interact directly with mitochondria, facilitating the regulation of mitochondrial gene expression [51]. This targeted delivery system allows cancer cells to hijack the metabolic and apoptotic machinery of the TME with remarkable precision.

## 3. The EV-miRNA-Mitochondria Axis of Oncogenesis

In the context of malignancy, MIPS is systematically hijacked by EV-miRNAs that act as remote epigenetic controllers. Unlike direct genetic mutations, MIPS allows a primary tumor to modulate the bioenergetic and apoptotic thresholds of neighboring and distant cells, effectively turning the TME into a supportive system for growth and invasion [1,5,20].

### 3.1. Bioenergetic Reprogramming (The Warburg and Reverse Warburg Symbiosis)

While normal cells rely on OXPHOS for energy production, cancer cells utilize EV-delivered bioactive cargo to enforce metabolic plasticity, often shifting energy production to aerobic glycolysis even in oxygen-rich environments [9,52]. Importantly, the EV-miRNA-mitochondria axis is not exclusively oncogenic; it exhibits a functional duality where specific EVs can also deliver tumor-suppressive miRNAs to drive anti-tumor mitochondrial reprogramming [53]. For instance, the EV-mediated transfer of miRNAs such as let-7a and miR-126 can actively suppress OXPHOS and alter cellular metabolism to halt tumor progression [54]. Furthermore, EVs delivering miR-27b or mesenchymal stem cell-derived miR-484 can severely disrupt mitochondrial activity in target cancer cells, leading to lethal ROS accumulation, bioenergetic exhaustion, and mitophagy-mediated apoptosis [8,55].

#### 3.1.1. Standard Warburg and the ETC Impairment

It has been shown that hypoxic tumor-derived EVs are highly enriched in miR-210, which directly impairs mitochondrial respiration in recipient cells [9,20]. miR-210 binds to the 3′-UTR of the NDUFA4, a subunit of Complex I, and the SDHD, a subunit of Complex II, leading to impairment of the ETC, which ultimately leads to a collapse of the mitochondrial membrane potential (MMP) and a mandatory shift toward glycolysis to maintain ATP levels [8,31]. Similarly, EV-miR-126 has been shown to inhibit ATP citrate lyase (ACL), disrupting the generation of cytosolic acetyl-CoA. This blockade restricts the availability of precursors for fatty acid biosynthesis, forcing a metabolic shift toward oxidative energy production that ultimately suppresses tumor malignancy and progression [56]. Importantly, the downstream consequences of EV-miR-210 are highly dependent on the cellular context. While it targets ETC components in stromal fibroblasts to force a glycolytic shift, the same miRNA alternatively targets inhibitors like Ephrin-A3 (EFNA3) when internalized by endothelial cells, thereby promoting angiogenesis rather than purely altering bioenergetics [3].

#### 3.1.2. The Reverse Warburg Effect and Metabolic Coupling

Cancer cells often co-opt stromal cells, particularly cancer-associated fibroblasts (CAFs) and adipocytes, to induce a “Reverse Warburg Effect” [57]. In this scenario, cancer cells secrete EVs containing miRNAs like miR-155 or miR-210 that force CAFs to undergo glycolysis and secrete energy-rich metabolites, such as lactate and pyruvate [11]. These metabolites are then imported by the tumor cells to fuel their own TCA cycle, creating a parasitic metabolic symbiosis that optimizes bioenergetics to sustain tumor survival and promote progression [9,11].

#### 3.1.3. Nutrient Sequestration

Mills et al. [11] have made a unique observation where metastatic breast cancer cells secrete miR-122 in vHD-EVs to reprogram the glucose metabolism of niche cells [20]. By targeting Pyruvate Kinase (PKM) and Glucose Transporter 1 (GLUT1) in lung fibroblasts and astrocytes, miR-122 suppresses their glucose utilization, thereby increasing nutrient availability for arriving metastatic “seed” cells [20]. Additionally, mesenchymal stem cell (MSC)-derived EV-cargo, such as miR-484, can function as a metabolic disruptor in pancreatic cancer [8]. By deactivating the Wnt/MAPK signaling pathway, miR-484 causes an upsurge in ROS and a significant drop in ATP levels, thereby inhibiting the proliferation of malignant cells through bioenergetic exhaustion [8].

### 3.2. Subverting Mitochondrial Dynamics: Fission, Fusion, and Motility

Mitochondria are not static organelles; there is a dynamic balance between continuous fission (fragmentation) and fusion (merging) to maintain regular cellular function [57]. At the molecular level, mitochondrial fusion is driven by Mitofusins (Mfn1 and Mfn2) and Optic Atrophy 1 (Opa-1) to form interconnected networks that maximize OXPHOS and protect against apoptosis. Conversely, fission is mediated by the dynamin-related GTPase Dynamin-related protein 1 (Drp1), which pinches mitochondria into smaller units essential for cellular transport and mitophagy. In cancer, dysregulating these processes allows tumor cells to alter their metabolic profiles and evade apoptosis [9]. EV-miRNAs exploit this balance to facilitate tumor motility and metastasis.

#### 3.2.1. Mitochondrial Fission-Driven Metastasis

A shift toward mitochondrial fission generates smaller, highly mobile mitochondria that can be easily distributed to areas of high cellular energy demand, facilitating tumor motility and the epithelial-to-mesenchymal transition (EMT) [29]. Hypoxic breast cancer cells-derived EVs promote Drp1 phosphorylation at the Ser616 site in recipient epithelial cells, triggering significant mitochondrial fission. These small, fragmented mitochondria are more easily transported to the cell’s cortical cytoskeleton, providing the localized ATP required for cytoskeletal remodeling and migration, which directly drives cancer cell invasion [9].

Beyond direct Drp1 activation, cells in the TME can also supply the actual fission machinery and metabolic substrates via EVs to activate this process. For example, adipocyte-derived EVs have been shown to transfer fatty acids, fatty acid oxidation (FAO) enzymes, and specific mitochondrial fission regulators, namely FIS1 and Opa-1, directly to melanoma cells [9]. This cargo enhances FAO, which subsequently stimulates robust mitochondrial fission and the redistribution of these smaller mitochondria to cellular protrusions, markedly increasing the tumor’s invasive capacity [9]. Furthermore, the hypoxic conditions typical of solid tumors trigger the production of ROS, which acts as an additional stress signal to upregulate mitochondrial fission; this not only drives metastasis but also serves as an adaptive mechanism to confer chemoresistance [51].

#### 3.2.2. Mitochondrial Fusion Inhibition and Aggressiveness

It has been shown that in urological and pancreatic malignancies, CAF-derived EVs carrying miR-106b target Mfn2 in recipient cells [58]. The loss of Mfn2 inhibits mitochondrial fusion, leading to a fragmented mitochondrial network that is associated with increased invasive behavior and resistance to apoptosis. Because highly fused mitochondrial networks delay the release of cytochrome c, this EV-mediated loss of fusion proteins successfully alters the cell’s apoptotic threshold to support survival. Conversely, it has also been observed that EV-transferred miRNAs stabilize mitochondrial fusion factors like Opa1 in recipient cells to prevent mitophagy and ensure a robust mitochondrial network under the stress of chemotherapy, conferring significant drug resistance to the cancer cells [57].

### 3.3. Evasion of the Intrinsic Apoptotic Pathway

The threshold for programmed cell death is regulated by the Bcl-2 family of proteins, which is also expressed on the outer mitochondrial membrane [59]. EV-miRNAs, such as miR-21, have been observed to frequently raise the threshold by targeting pro-apoptotic factors (e.g., PDCD4, PTEN, Bax) or upregulating anti-apoptotic Bcl-2, conferring broad chemoresistance [60,61].

#### 3.3.1. Anti-Apoptotic Shielding

EV cargo oncomiRs (oncogenic miRNAs that promote tumor development by silencing tumor-suppressor genes) [35], like miR-21 and miR-221/222, are master regulators of the evasion of intrinsic apoptosis [29,61]. miR-21 binds to the 3′-UTR of pro-apoptotic genes such as PDCD4 and PTEN, preventing the activation of Bax and its translocation into the mitochondrial outer membrane. This effectively blocks the release of cytochrome c and the subsequent initiation of cell death [5,9].

#### 3.3.2. Raising the Resistance Threshold

It has been postulated that in pancreatic and colorectal cancers, the downregulation of EV-miR-34a leads to a significant increase in anti-apoptotic protein Bcl-2 expression within the tumor cells. The changes in Bcl-2 levels ultimately lead to an increased mitochondrial apoptotic threshold, effectively creating a survival barrier that confers resistance to Cisplatin and gemcitabine [8,60]. Similarly, the downregulation of EV-miR-125a-5p promotes survival by relieving the suppression of the anti-apoptotic effectors BCL2L12 and MCL1, essentially ‘locking’ the recipient cancer cell in a survival state [62].

#### 3.3.3. Targeting Transcriptional Regulators

In ovarian cancer, MSC-derived EVs carrying miR-424 have been found to target MYB, a transcription factor. Downregulating MYB, miR-424 inhibits VEGF/VEGFR signaling and thereby suppresses both tumorigenesis and pathological angiogenesis [63]. Conversely, in colorectal cancer, tumor-derived EVs deliver miR-25-3p to endothelial cells, where it targets the transcription factors KLF2 and KLF4 to disrupt the endothelial barrier and promote vascular permeability [63]. EVs from CAF transfer miR-135b-5p, which downregulates the transcription factor FOXO1, resulting in enhanced endothelial cell migration and tube formation [63]. Furthermore, in renal cell carcinoma, the downregulation of EV-miR-549a relieves the suppression of HIF1α, a master transcriptional regulator of hypoxia, thereby facilitating neovascularization and metastasis [58].

### 3.4. Structural Sabotage: mtDNA and Organelle Transfer

Zhang et al. [51] have identified that beyond the transfer of regulatory miRNAs, EVs serve as vehicles for the horizontal transfer of the mitochondrial genome (mtDNA) and even entire functional organelles [39]. This transfer effectively restores OXPHOS in metabolically compromised recipient cells, allowing them to rescue bioenergetics and, in the context of cancer, exit metabolic dormancy to resume progression [64]. Mechanistically, these processes serve distinct biological roles in tumor progression. While the transfer of whole, functional mitochondria primarily provides a direct physical rescue to replace damaged machinery and restore OXPHOS, the transfer of mtDNA fragments frequently acts as a remote signaling mechanism, functioning as damage-associated molecular patterns (DAMPs) that activate pathways like TLR9 to polarize the immune microenvironment toward an immunosuppressive state [9].

#### 3.4.1. Horizontal Mitochondrial Transfer

It has been validated that MSCs- and tumor-activated stromal cells can export whole, respiration-competent mitochondria within large EVs to rescue the bioenergetics of cells with damaged or depleted mitochondria [65,66]. Further, in hormone-resistant breast cancer and leukemia, the receipt of these functional mitochondria allows cancer cells to exit dormancy and resume rapid proliferation despite treatment [51].

#### 3.4.2. “Mitovesicles” and mtDNA Propagation

**Figure 1:**
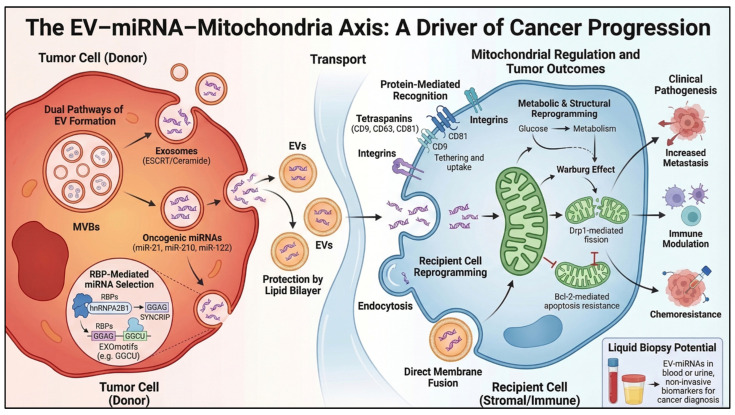
**The EV—miRNA—mitochondria axis in cancer progression.** Tumor cell-derived Extracellular Vesicles (EVs), including exosomes and microvesicles, through ESCRT-dependent or ceramide-mediated pathways. Selectively packaged oncogenic miRNAs are transported within EVs and delivered to recipient stromal or tumor cells via endocytosis or membrane fusion. Upon internalization, EV-derived miRNAs regulate mitochondrial function and dynamics, promoting metabolic reprogramming consistent with the Warburg effect, resistance to apoptosis, and enhanced tumor growth, metastasis, and therapy resistance. Circulating EV-miRNAs also represent promising non-invasive biomarkers for cancer diagnosis and monitoring. Image Created Using Google Draw.io (https://www.drawio.com/).

The discovery of “mitovesicles”, a specialized EV sub-population of mitochondrial origin, reveals that cells can export mitochondrial components like VDAC, COX-IV, and double-stranded mtDNA fragments directly into the extracellular space [67]. Beyond this specific subtype, various stress conditions trigger the release of EVs carrying mitochondrial cargo with potent immunomodulatory effects. For instance, activated monocytes and platelets release mitochondria-containing EVs that induce Type I interferon and TNF responses in endothelial cells [68,69]. Mitochondrial LON protease promotes the secretion of mtDNA-enriched EVs, which activate TLR9 signaling in macrophages to induce an immunosuppressive M2 phenotype [70]. Furthermore, T-cells have been observed to transfer mtDNA via EVs to dendritic cells, triggering an antiviral immune response [71]. Collectively, these vesicles possess their own enzymatic activity and can alter the inflammatory landscape of recipient immune cells through TLR9 or cGAS-STING signaling pathways. As shown in Figure 1, a brief overview of the EV—miRNA—mitochondria axis in cancer progression.

Because EV-miRNAs act as post-transcriptional regulators rather than inducing permanent genetic changes, their effects on recipient mitochondrial dynamics and apoptosis are inherently transient. Kinetic studies demonstrate that internalized EV-RNAs progressively degrade over time, indicating that the maintenance of this metabolic reprogramming relies entirely on a continuous supply of tumor-derived EVs [72].

## 4. Clinical Implications of EV-Mediated Mitochondrial Reprogramming in Malignancies

While the fundamental machinery of EV biogenesis and mitochondrial reprogramming is conserved, the specific miRNAs utilized and the functional outcomes vary significantly across different malignancies. Several studies have identified the molecular mechanism by which the EV-miRNA-mitochondria axis drives pathogenesis in pancreatic, neurological, breast, hematologic, and urological cancers. Table 1 delineates this in various cancer types.

### 4.1. Pancreatic Cancer: Wnt/MAPK Pathway

Pancreatic Ductal Adenocarcinoma (PDAC) is characterized by a dense, hypoxic desmoplastic stroma. Recent pharmacological research has revealed a crosstalk where stromal cell-derived EVs deliver miR-484 to tumor cells; this interaction disrupts mitochondrial metabolism through the activation of the Wnt/MAPK pathway, resulting in bioenergetic exhaustion and tumor suppression [8]. While many tumor-derived EVs promote progression, EVs derived from human bone marrow MSCs (hBMSCs) can exert therapeutic effects by dismantling mitochondrial metabolism. Specifically, in pancreatic cancer, hBMSC-derived EVs deliver miR-484 to tumor cells, which directly deactivates the Wnt/MAPK signaling pathway. This targeted deactivation disrupts mitochondrial function, leading to a significant decrease in ATP production and a lethal accumulation of ROS. In vivo analyses confirmed that this EV-mediated transfer shrinks tumor size and weight, while markedly suppressing the expression of essential mitochondrial proteins, such as HSP60 and SDHA, alongside various electron transport chain genes [8].

#### 4.1.1. Altering the Respiratory Energy Reserve

Targeting mitochondrial metabolism has emerged as a highly attractive strategy for cancer therapy, particularly in pancreatic cancer, where suppressing mitochondrial function is known to severely repress tumor cell proliferation. The mitochondrial suppression directly affects the mitochondrial bioenergetics in the cancer cells. As a promising therapeutic application, human bone marrow mesenchymal stem cell (hBMSC)-derived exosomes can be utilized to deliver tumor-suppressive miRNAs, such as miR-484, directly to pancreatic tumors [8]. This disruption of mitochondrial metabolism downregulates the expression of key ETC genes, such as ND1, ND2, ATP5, Cytb and fatty acid oxidation regulators, like PPAR. Downregulation of ETC genes results in a significant decrease in mitochondrial ATP production and a concurrent increase in mitochondrial ROS production. This ROS overload activates apoptosis and inhibits tumor growth, in vivo, highlighting how the EV-mitochondria axis can be hijacked therapeutically to induce metabolic exhaustion and mitigate malignant transformation in cancer cells [8]. The OXPHOS can increase the survival of pancreatic cancer cells and impair the therapeutic importance of KRAS-ablation therapy. The clinical-grade engineered hBMSC-derived exosomes with the ability to target oncogenic Kras (iExosomes) confirm suppression of oncogenic Kras and an increase in the survival of several mouse models with pancreatic cancer [80].

#### 4.1.2. Activation of Pro-Tumorigenic Fibroblast Loop

It has been shown that the pancreatic cancer cells-derived EVs containing miR-155, which involves reprogramming of normal fibroblasts into CAFs. These CAFs then secrete EVs that protect the tumor cells from oxidative stress by upregulating superoxide dismutase (SOD2) and catalase (CAT), effectively shielding the tumor mitochondria from gemcitabine-induced ROS [17].

### 4.2. Glioblastoma (GBM): The Hypoxic Neural Niche

It has been shown that the GBM cells can alter the brain microenvironment via EV biogenesis, utilizing specific RBPs to sort miRNAs that alter mitochondrial dynamics and immune responses [29]. Several factors have been revealed that can selectively load miRNAs to alter the tumor microenvironment.

#### 4.2.1. Mex-3 RNA Binding Family Member C (MEX3C) Can Selectively Load miR-451a into the EVs

In GBM cells, MEX3C and RBP interact with the clathrin adaptor protein AP-2 to selectively sort miR-451a into EVs. miR-451a is a key regulator of the LKB1/AMPK pathway, which governs metabolic adaptation to glucose deprivation. By exporting miR-451a, or transferring it to microenvironmental cells, GBM cells modulate the metabolic stress response of the entire niche [29].

#### 4.2.2. Hypoxia and Immune Evasion

It has been identified that hypoxic glioma cells release EVs enriched with miR-1246 expression. Once these EVs are taken up by tumor-associated macrophages (TAMs), miR-1246 directly binds to the 3′-UTR of TERF2IP mRNA [29,81]. The inhibition of TERF2IP activates the STAT3 and NF-κB pathways, polarizing macrophages toward the immunosuppressive M2 phenotype. These M2 macrophages then support tumor growth by secreting pro-angiogenic factors and suppressing T-cell mitochondrial function [29,82].

#### 4.2.3. Development of Temozolomide (TMZ) Resistance

Chemoresistance in GBM is frequently mediated by EV-miRNAs that protect mitochondrial integrity. miR-151a is transferred from TMZ-resistant cells to sensitive cells via EVs [83]. It targets X-ray repair cross-complementing 4 (XRCC4), modulating DNA repair mechanisms that are energetically dependent on mitochondrial ATP [29]. Additionally, GBM-secreted EVs carry miR-21, which targets apoptotic regulators to prevent mitochondrial outer membrane permeabilization (MOMP) induced by chemotherapy [29].

### 4.3. Breast Cancer: Metabolic Coupling and Chemoresistance

As described earlier, metastatic breast cancer cells (e.g., MDA-MB-231) secrete a specialized subpopulation of vHD enriched with miR-122 [21]. Metabolic regulation occurs upon reaching distant organs, such as the lung or brain, where miR-122 gets internalized from the vHD-EVs by resident stromal cells, mainly fibroblasts and astrocytes. The miRNA targets PKM and GLUT1, suppressing glucose uptake in these niche cells to increase nutrient availability for the arriving tumor cells [84]. By suppressing stromal metabolism, the tumor effectively increases the extracellular glucose available for its own mitochondria, fueling the high bioenergetic costs of colonization.

It has been revealed that the EVs function as “resistance vectors” in breast cancer therapy. Adriamycin/Docetaxel-resistant breast cancer cells secrete EVs containing miR-221/222 and miR-1246 [61]. Upon transfer to neighboring, initially chemo-sensitive breast cancer cells, miR-221/222 binds to the 3′-UTR of p53 upregulated modulator of apoptosis (PUMA) and p27 mRNAs, blocking the intrinsic mitochondrial apoptotic pathway and conferring resistance [5]. Furthermore, serum EV-miR-210, a hypoxia-induced miRNA, level correlates with trastuzumab resistance and tumor progression by modulating mitochondrial respiration and angiogenesis [20]. Specifically, EV-delivered miR-210 promotes vascularization by targeting and downregulating the angiogenic inhibitor Ephrin-A3 (EFNA3) in endothelial cells, thereby enhancing VEGF signaling and tube formation [1,4,18,68].

### 4.4. Hematologic Malignancies: Remodeling the Bone Marrow

In leukemia and lymphoma, it has been found that EVs traffic between blast cells and the bone marrow stroma, creating a sanctuary for cancer stem cells [85,86]. Several studies have identified that the Acute Myeloid Leukemia (AML) cells release EVs that contain miR-155 and miR-125b [86,87]. Bazi et al. [2] have demonstrated that miR-155 and miR-125b are transferred to hematopoietic stem cells (HSCs) and stromal cells through EVs, where they suppress c-MYB and BCL2 Antagonist/Killer 1 (BAK1). This suppression prevents mitochondrial apoptosis in the leukemic cells while impairing the differentiation and fitness of healthy HSCs. Additionally, AML-derived EVs carrying miR-1246 target the LRIG1/STAT3 axis in HSCs, reducing protein synthesis and mitochondrial activity, which forces stem cells into a quiescent, drug-resistant stage [2]. EVs from chemo-resistant AML cells can horizontally transfer resistance to sensitive cells by delivering miR-19b and miR-20a, which upregulate the multidrug resistance protein 1 (MRP1) and enhance drug efflux capacity [88].

The interaction between Multiple Myeloma (MM) cells and bone marrow MSCs (BM-MSCs) is bi-directional. It has been demonstrated that under oxidative stress, BM-MSCs can transfer whole functional mitochondria to MM cells via large EVs or tunneling nanotubes. This transfer reduces mitochondrial ROS in the cancer cells and restores OXPHOS, directly counteracting the effects of proteasome inhibitors like bortezomib [89]. In another study, Bazi et al. [2] have shown that the MM-derived EVs transfer miR-146a to MSCs, triggering the secretion of pro-inflammatory cytokines that support tumor growth and osteolysis. Again, under hypoxic conditions, MM cells release EVs enriched with miR-135b that are internalized by endothelial cells; this transfer translationally inhibits Factor-Inhibiting HIF-1 (FIH-1) to promote endothelial tube formation and angiogenesis [90]. Furthermore, MM-derived EVs delivering miR-16 have been found to target the IKK/complex in monocytes, driving their differentiation into immunosuppressive M2 macrophages to support immune evasion [91].

### 4.5. Gynecological, Urological and Ovarian Cancer: Angiogenesis

MSC-derived EVs have shown therapeutic potential in ovarian cancer by regulating the miR-424/MYB signaling pathway. MSC-EVs deliver miR-424 to ovarian cancer cells and endothelial cells. miR-424 directly binds to the 3′-UTR of MYB [92]. Downregulation of MYB leads to a marked reduction in VEGF expression. This inhibits endothelial tube formation (angiogenesis) and suppresses tumor cell proliferation by starving the tumor of oxygen and nutrients [92]. In renal cell carcinoma (RCC), it has been identified that the circulating EVs are enriched with miR-210 [93,94,95]. miR-210 levels in serum EVs correlate with the hypoxic status of the tumor. It has been shown that miR-210 directly binds to the iron-sulfur cluster assembly proteins (ISCU) mRNA in recipient cells, resulting in Complex I of the ETC being dysfunctional and enforcing a glycolytic shift that supports survival in the avascular tumor core [20].

### 4.6. Clinical Translation: EV-miRNAs as Non-Invasive Biomarkers

The mechanistic role of EV-miRNAs in subverting mitochondrial function and driving angiogenesis translates directly into clinical utility. Because these hypoxia- and metabolism-altering miRNAs are selectively enriched in EVs during tumor progression, their detection in biofluids offers a clinically relevant diagnostic tool. For example, the EV-miR-210, which drives the glycolytic shift in RCC by targeting mitochondrial Complex I, is readily detectable in-patient serum and serves as a highly sensitive biomarker for clear cell RCC [96]. Furthermore, this diagnostic potential extends beyond serum to other biofluids; the presence of specific miRNAs, such as miR-30c-5p in urinary EVs, provides a completely non-invasive method to successfully distinguish clear cell RCC from benign conditions with near 100% specificity [52,81]. Table 2 summarizes cancer-specific EV-miRNA interactions with mitochondrial pathways, highlighting their molecular effects and associated clinical outcomes.

## 5. Methodological Standards for Clinical Validation of EV-miRNA Biomarkers

While EV-miRNAs hold immense promise as non-invasive biomarkers for tracking tumor progression and metabolic shifts, their clinical validation requires overcoming significant technical challenges and establishing rigorous methodological standards [4]. A primary hurdle is the lack of standardized isolation methods, which can lead to the co-isolation of non-vesicular proteins and lipoproteins [35]. Unfortunately, most current miRNA biomarkers are unlikely to have much clinical utility. As we and others have shown, strategies for identifying miRNA biomarkers in the first wave of discovery were inadequate and challenged by variable isolation methods, non-standard analysis designs, and an underappreciation of the cellular source of an miRNA [97]. To ensure clinical reliability, accurate pre-analytical processing is essential. For instance, utilizing plasma rather than serum is often preferred to avoid contamination from coagulation-associated EVs released by platelets and lysed blood cells [35]. Furthermore, because EVs can associate with protein complexes bound to free-circulating miRNAs, treatments utilizing proteinase K and RNase A prior to miRNA extraction are heavily recommended during the biomarker discovery and clinical validation phases to remove extravesicular contamination [35]. While these enzymatic treatments may be too cumbersome for routine, high-throughput clinical diagnostics, they are essential for establishing initial biomarker accuracy; failure to implement these steps during validation can result in up to a 70% over-estimation of EV-miRNA quantities in patient plasma due to externally bound contaminants. Ensuring the analyzed signature is genuinely encapsulated within the EV [4]. Analytical variability poses another major challenge, as current RNA quality control standards were originally optimized for cellular RNA and do not accurately represent the unique RNA cargo typically found in small EVs. Furthermore, achieving effective RNA quantification requires the establishment of robust biological normalization controls (such as stable small non-coding transcripts) to overcome technical variability before downstream computational processing [4]. Therefore, rigorous normalization strategies must be implemented. This includes establishing optimal endogenous reference miRNAs (such as miR-16-5p, miR-423-3p, or miR-191-5p) or utilizing exogenous normalizers (e.g., spike-in cel-miR-39) to reduce technical noise and inter-individual variability across clinical samples [35]. Finally, to ensure reproducibility and facilitate successful clinical translation, studies must adhere to universally established reporting frameworks, such as the MISEV 2023 (Minimal Information for Studies of Extracellular Vesicles) guidelines, which provide foundational criteria for EV isolation and characterization [35].

## 6. Conclusions

By elucidating the specific mechanisms through which EV-carried miRNAs regulate mitochondrial pathways in the recipient cells, this review provides a comprehensive insight into the interplay between EV-mediated miRNA transport and mitochondrial function in oncogenesis. Understanding how these miRNAs influence mitochondrial dynamics, bioenergetics, and apoptosis provides critical information about how tumor cells adapt, survive, and proliferate within hostile microenvironments. This knowledge not only clarifies the role of the mitochondrial pathway as an oncogenic axis but also underscores the significance of EVs as vehicles for molecular communication and regulation in cancer progression. Furthermore, the review highlights the potential of EV-carried miRNAs in clinical applications. Their stability in biological fluids and specificity for disease states make them attractive candidates for novel diagnostic biomarkers that could allow for non-invasive cancer detection, monitoring, and prognosis. In addition, uncovering the functional consequences of miRNA-mediated mitochondrial dysregulation opens new avenues for therapeutic intervention, including targeted delivery of anti-miRs or mimics via EVs to restore mitochondrial homeostasis and inhibit tumor growth. The clinical translation of EV-based miRNA therapeutics must overcome several formidable barriers. Systemically administered EVs are frequently subjected to rapid clearance by the mononuclear phagocyte system, leading to poor tumor biodistribution and potential off-target effects that necessitate advanced surface engineering to improve tumor tropism [3,98]. Furthermore, while native EVs generally exhibit low immunogenicity, potential immune and inflammatory reactions to repeated administrations of engineered or allogeneic EVs remain a clinical safety concern [5]. Finally, large-scale clinical deployment is currently hindered by significant manufacturing challenges, including the lack of standardized isolation protocols, low vesicle yields, batch-to-batch inconsistency, and suboptimal miRNA loading efficiencies [4].

Overall, this review seeks to integrate current knowledge, identify critical gaps, and propose future directions for research, with the goal of translating these mechanistic insights into innovative strategies for cancer diagnosis and treatment.

## Figures and Tables

**Table 1:** EV-miRNA functions based on tumor-promoting vs. tumor-suppressive roles.

Functional Role	EV-miRNA	Cancer Type	Cellular Source (Donor)	Target Cell (Recipient)	Key Mechanism and Biological Outcome	References
Tumor-Promoting	miR-105	Breast	Metastatic breast cancer cells	Endothelial cells	Destroys ZO-1 and tight junctions, promoting vascular leakiness and distant metastasis.	[73]
	miR-155	Pancreatic	Pancreatic cancer cells	Normal fibroblasts	Converts normal fibroblasts into pro-tumorigenic cancer-associated fibroblasts (CAFs).	[74]
	miR-92a-3p	Colorectal (CRC)	Cancer-Associated Fibroblasts (CAFs)	CRC tumor cells	Promotes chemoresistance to 5-fluorouracil (5-FU) and oxaliplatin therapies.	[75]
	miR-21	Liver (HCC)/Breast	Tumor cells, Adipocytes	Hepatic stellate cells, Macrophages	Activates CAFs, drives macrophage M2 polarization, and stimulates angiogenesis.	[76]
Tumor-Suppressive	miR-484	Pancreatic	Human bone marrow MSCs (hBMSCs)	Pancreatic cancer cells	Deactivates the Wnt/MAPK pathway, causing ATP depletion, ROS accumulation, and tumor shrinkage.	[8]
	miR-34a-5p	Colorectal (CRC)	Mesenchymal stem cells (MSCs)	CRC tumor cells	Suppresses c-MYC/DNMT3a/PTEN axis, inhibiting tumor growth and epithelial–mesenchymal transition (EMT).	[77]
	miR-124-5p	Acute Myeloid Leukemia (AML)	Bone marrow MSCs (BMSCs)	Leukemic cells	Inhibits cell-cycle progression and induces apoptosis in AML cells.	[78]
	miR-655-3p	Esophageal (ESCC)	Human umbilical cord MSCs (hUCMSCs)	ESCC tumor cells	Inactivates HIF-1α via the LMO4/HDAC2 axis, significantly inhibiting liver metastasis.	[79]

**Table 2:** Cancer-specific EV-miRNA-mitochondria interactions.

Cancer Type	Key EV-miRNA	Mitochondrial/Molecular Effect	Clinical Outcome	Method	Citation
Pancreatic	miR-484	Suppresses Wnt/MAPK; Increases ROS; Decreases ATP	Tumor suppression; Metabolic exhaustion	Experimentally validated (in vitro assays and in vivo xenograft models)	[8]
Glioblastoma	miR-451a	Targets LKB1/AMPK; Metabolic stress adaptation	Glucose sensing; Niche remodeling	Experimentally validated (in vitro metabolic assays)	[29]
Breast	miR-122	Targets PKM/GLUT1; Suppresses stromal glucose uptake	Nutrient hijacking (Reverse Warburg)	Experimentally validated (in vivo pre-metastatic niche models)	[17]
Breast	miR-221/222	Targets PUMA/p27; Blocks mitochondrial apoptosis	Tamoxifen/Adriamycin Resistance	Experimentally validated (in vitro drug resistance and apoptosis assays)	[2,5]
AML	miR-125b	Targets BAK1; Inhibits apoptosis	Chemoresistance; HSC suppression	Clinical correlation and in vitro validation (Patient serum profiling and cell viability assays)	[2]
Ovarian	miR-424	Targets MYB; Downregulates VEGF	Inhibition of Angiogenesis	Experimentally validated (Dual-luciferase reporter assay and in vivo murine models)	[92]
Renal	miR-210	Targets ISCU/COX10; Inhibits ETC	Hypoxic adaptation; Glycolysis	Experimentally validated (in vitro hypoxia models)	[20]

## Data Availability

No new data were created or analyzed in this study. Data sharing is not applicable to this article.
